# Supplementary material for: Quercetin 3,5,7,3′,4′-pentamethyl ether from Kaempferia parviflora directly and effectively activates human SIRT1
Source: Commun Biol. 2021 Feb 19;4:209. doi: 10.1038/s42003-021-01705-1 (PMC7896056; doi:10.1038/s42003-021-01705-1)
Supplement: Supplementary file 1 — Supplementary Information [file 42003_2021_1705_MOESM1_ESM.pdf]

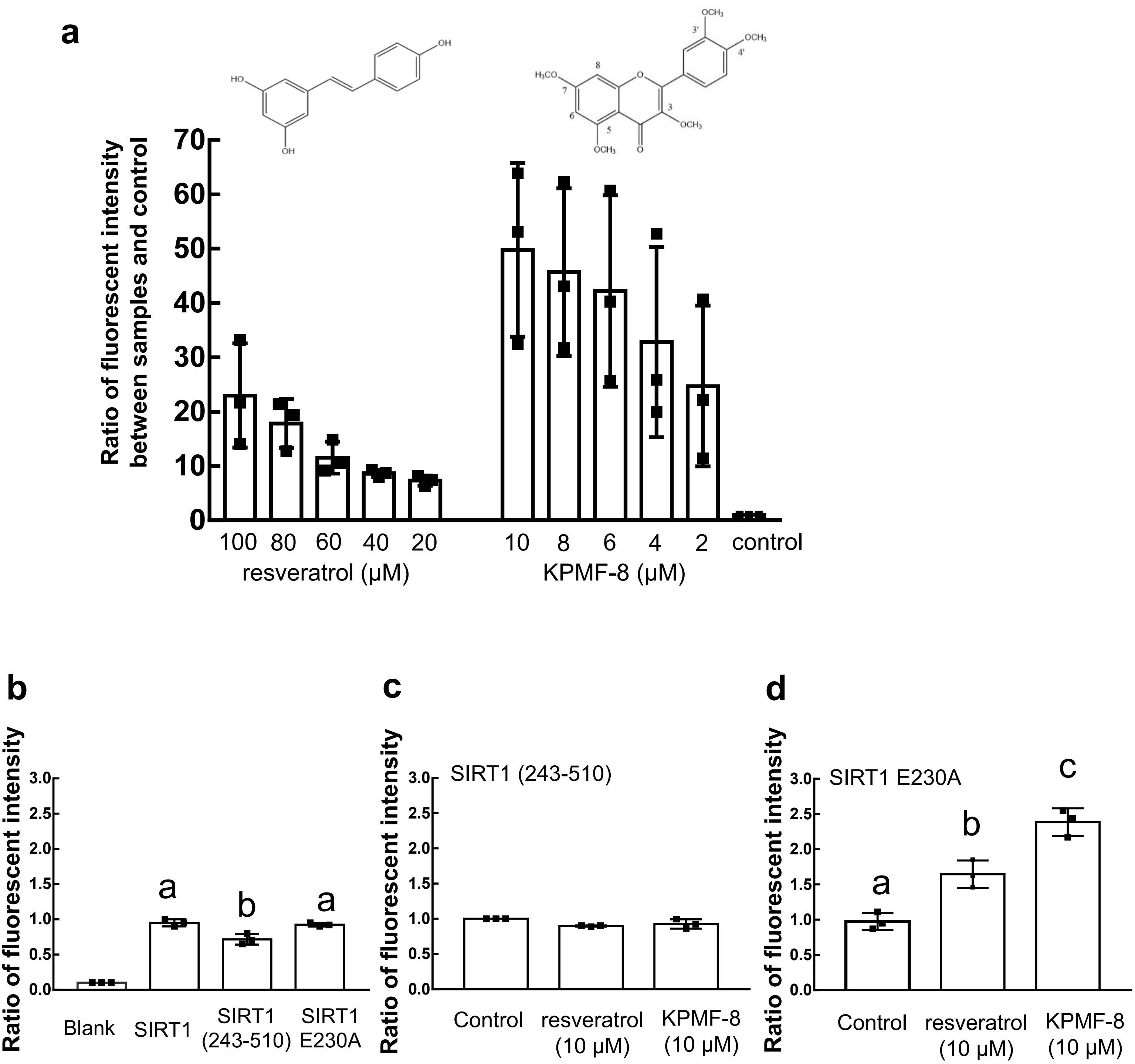

Supplementary Fig. 1 KPMF-8 and resveratrol stimulate SIRT1 activity *in vitro*. a Deacetylation of Ac-p53-AMC by recombinant SIRT1 in the presence of 2-10 μM KPMF-8 and 20-100 μM resveratrol. **b** Deacetylation of Ac-p53-AMC by recombinant full-length SIRT1, SIRT1 (243-510) and SIRT1 E230A. **c** Deacetylation of Ac-p53-AMC by SIRT1 (243-510) construct in the presence 10 μM resveratrol and 10 μM KPMF-8. **d** Deacetylation of Ac-p53-AMC by SIRT1 E230A mutant in the presence 10 μM resveratrol and 10 μM KPMF-8. Results are presented as sample/control fluorescence intensity ratio (Mean ± SD, n = 3).

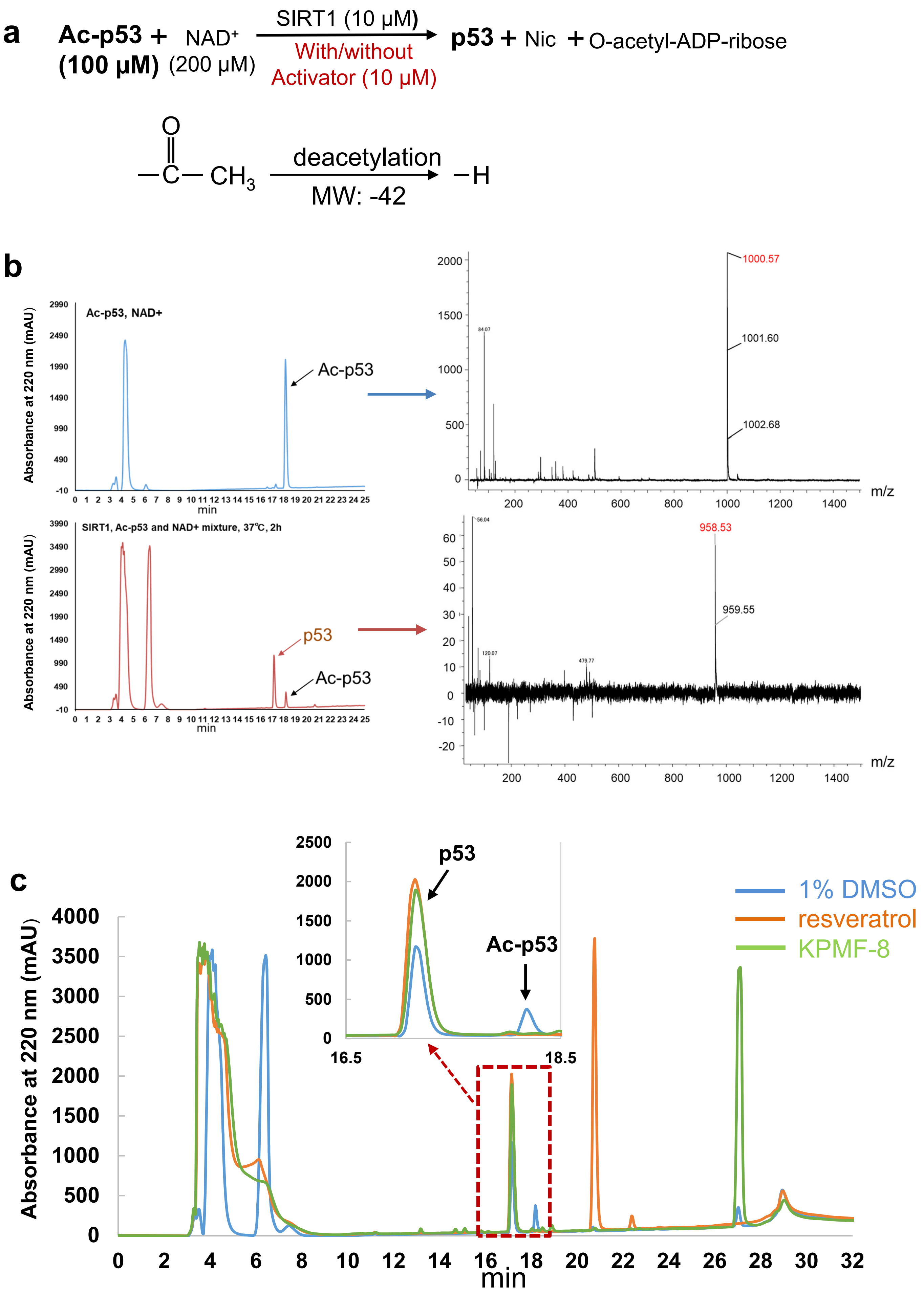

Supplementary Fig. 2 SIRT1 deacetylase activity measured by HPLC of native Ac-p53 peptide substrate and its deacetylated product. a Reaction scheme. **b** Identification of Ac-p53 peptide and deacetylated product by ESI-MS. **c** HPLC chromatograms of the SIRT1 reaction products at 37ºC for 2 h in the presence of 1% DMSO (blue), 10 μM resveratrol (orange) or 10 μM KPMF-8 (green).

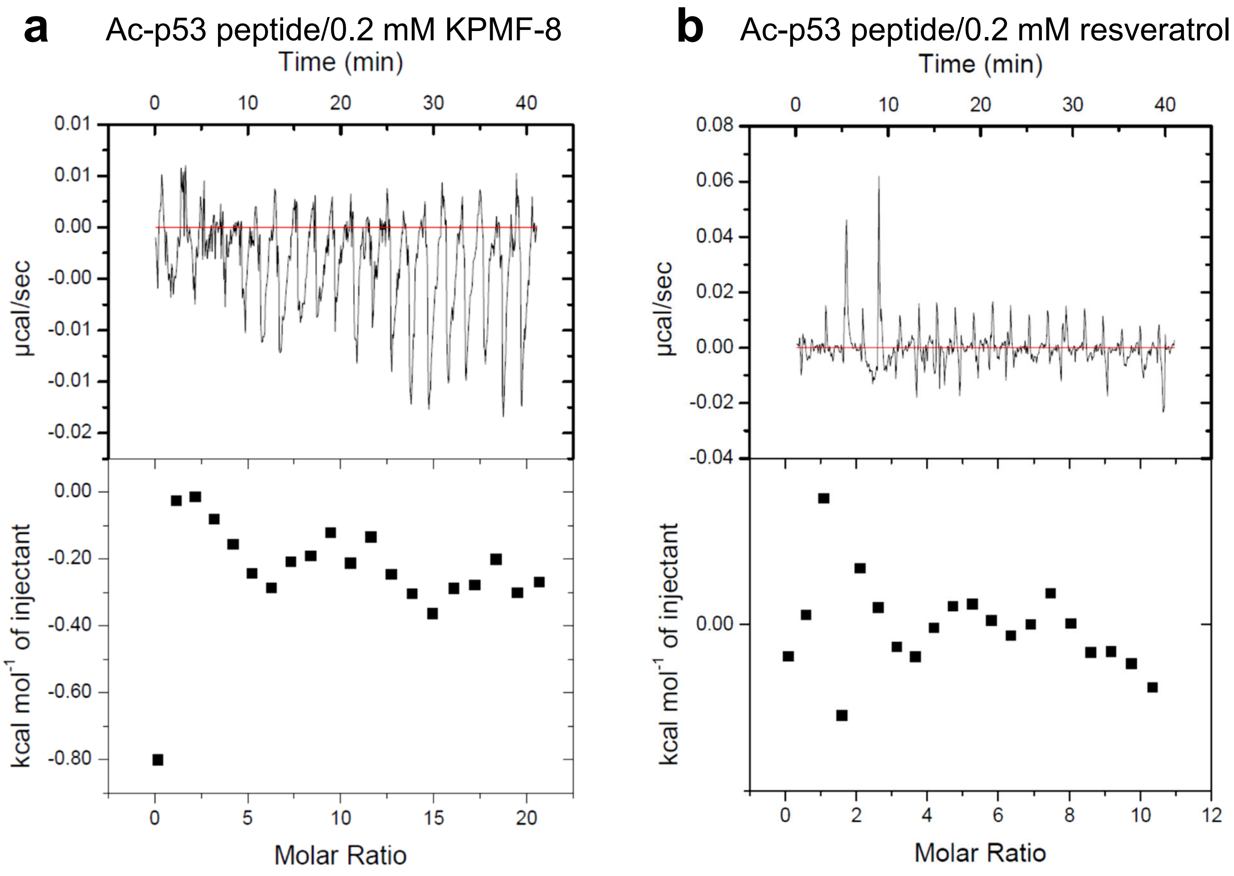

Supplementary Fig. 3 Binding of Ac-p53-derived peptide to KPMF-8 and resveratrol determined using ITC. a KPMF-8. **b** resveratrol. No affinity was observed between Ac-p53-derived peptide and KPMF-8 or resveratrol.

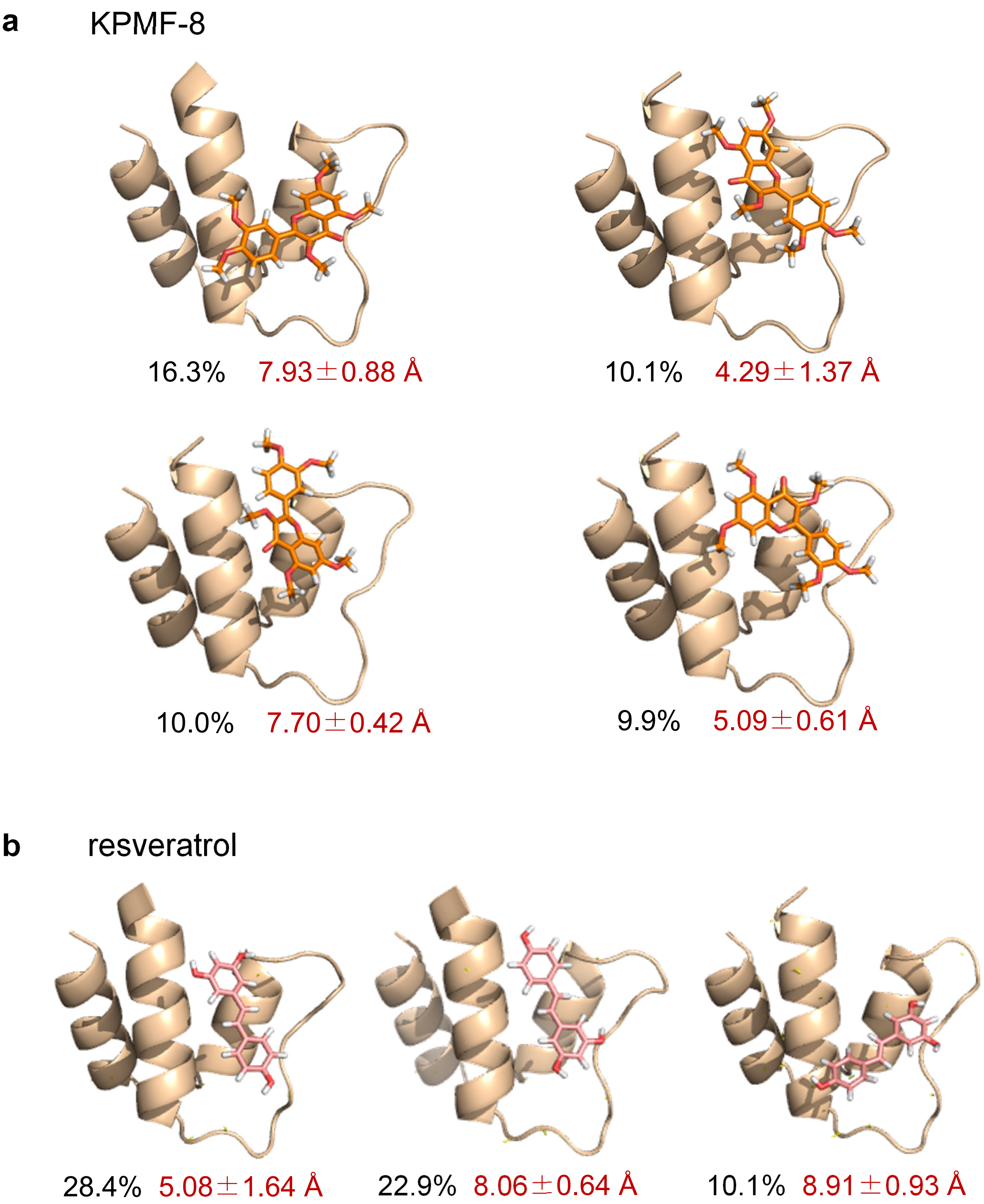

Supplementary Fig. 4 Representative structures of the clusters generated by the cluster analysis of the MD trajectories. a The SIRT1 NTD–KPMF-8 model. **b** The SIRT1 NTD–resveratrol model. Percentage shows the ratio of the number of structures grouped in each cluster to the total number of the structures in the MD trajectories. RMSD values (average ± standard deviation, shown in red) were obtained from the cluster analysis performed for the trajectories of all the runs.

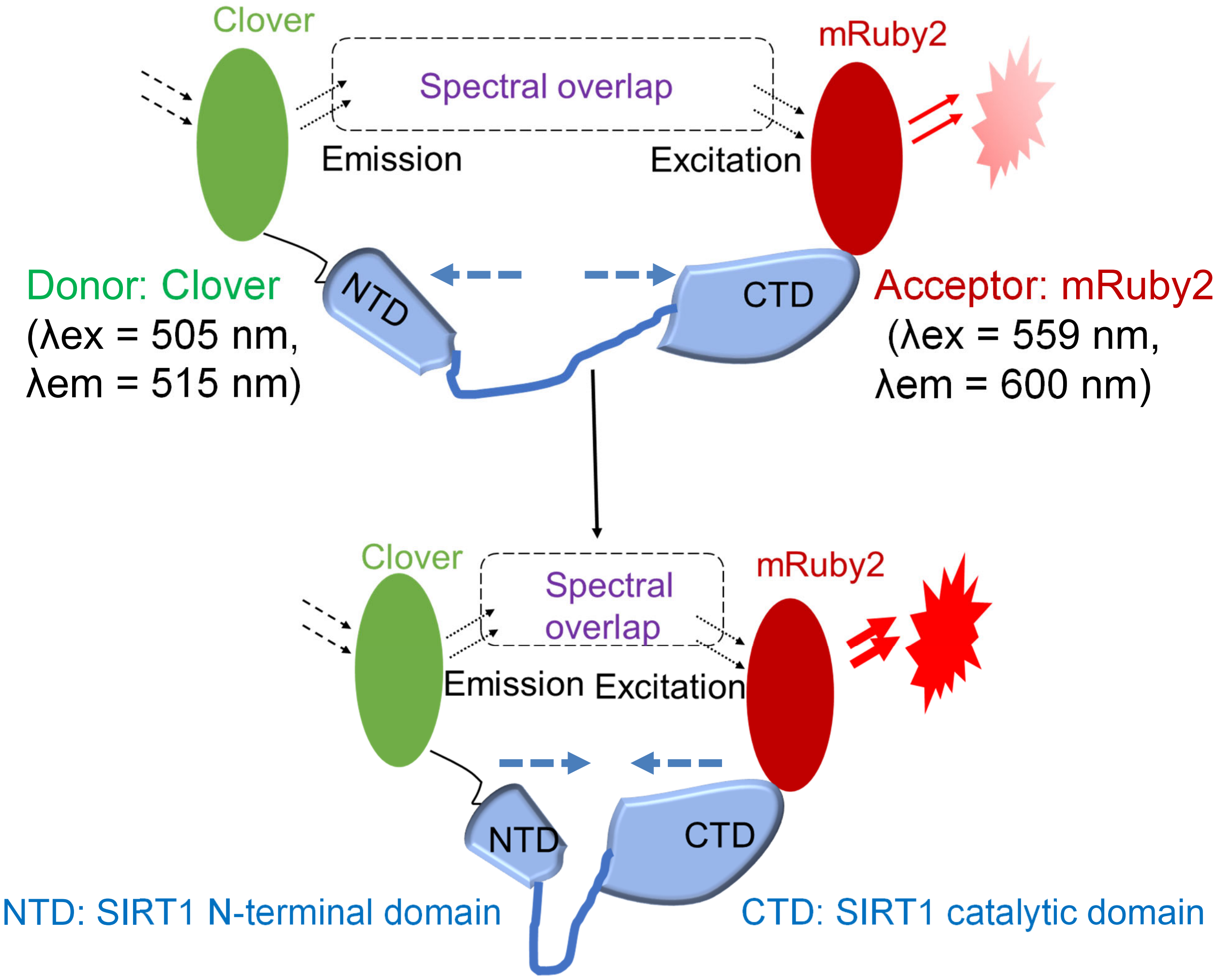

Supplementary Fig. 5 Schematic diagram of protein recombinant used for FRET assay. SIRT1 (183–510+GS+641–665) was inserted between two fluorescence proteins, Clover and mRuby2. Clover was conjoined to the N-terminus of SIRT1 as a donor, and mRuby2 was conjoined to the C-terminus of SIRT1 as an acceptor. Clover-mRuby2 allows FRET experiments with higher signal/background fluorescence than CFP and YFP. The FRET ratio (Int (mRuby2)/Int (Clover)) obtained from the intensity of the acceptor at acceptor emission and the donor at donor emission was used to detect comformational change(s) in SIRT1 protein.

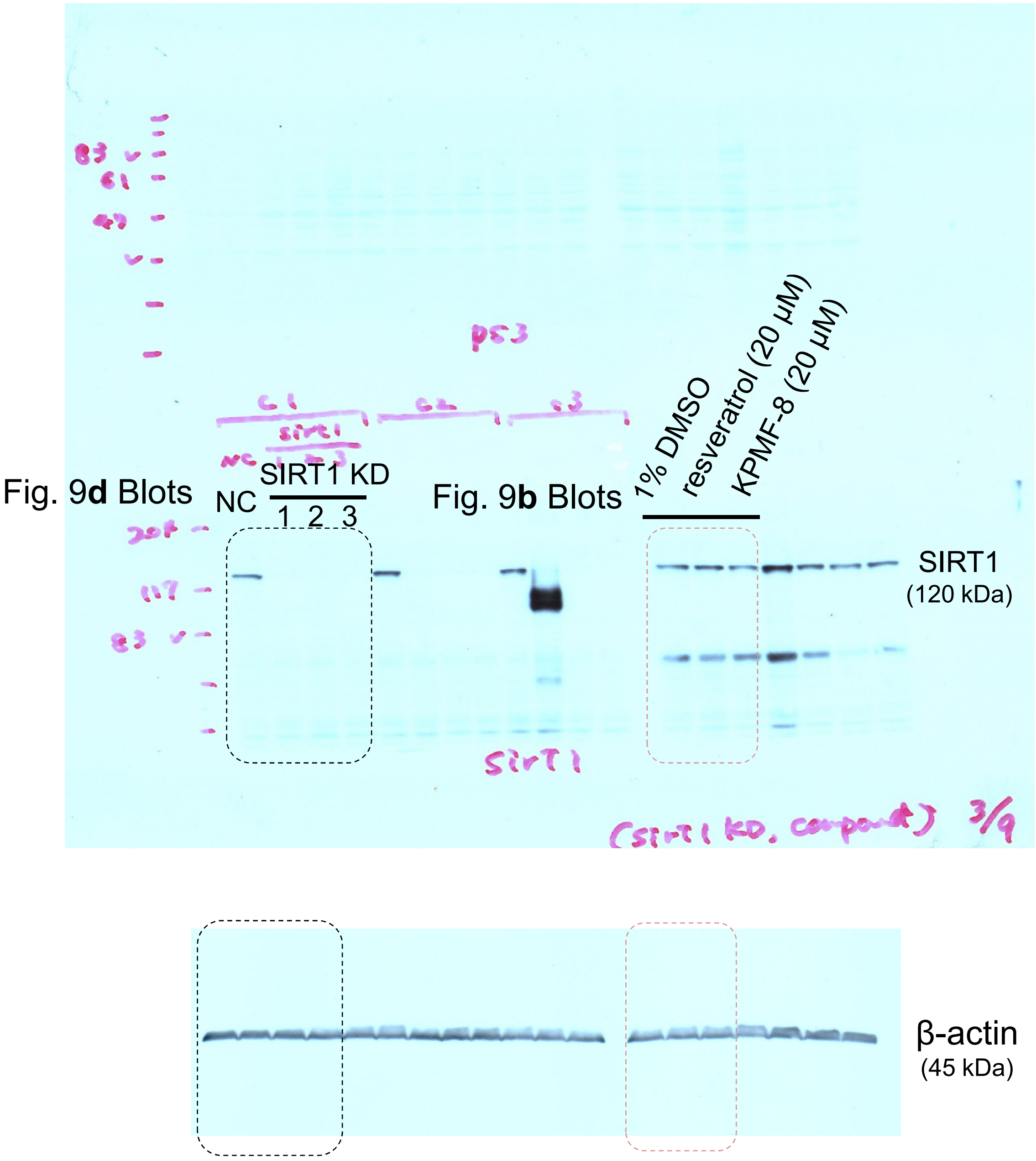

Supplementary Fig. 6 Full western blots illustrated in Fig. 9b, d. All lanes and antibodies used for staining are shown.
