# Supplementary material for: Quercetin 3,5,7,3′,4′-pentamethyl ether from Kaempferia parviflora directly and effectively activates human SIRT1
Source: Commun Biol. 2021 Feb 19;4:209. doi: 10.1038/s42003-021-01705-1 (PMC7896056; doi:10.1038/s42003-021-01705-1)
Supplement: Supplementary file 2 — Description of Additional Supplementary Files [file 42003_2021_1705_MOESM2_ESM.pdf]

Description of Additional Supplementary Files

File Name: Supplementary Data 1

Description: The input data, R codes and output data of the statistical analyses in Figs. 8, 9 and Supplementary Fig. 1.

File Name: Supplementary Data 2

Description: The source data underlying the graphs in Figs. 5, 7, 8, 9 and Supplementary Figs. 1 and 2.

File Name: Supplementary Data 3

Description: The atomic coordinates data used in Fig. 6 and Supplementary Fig. 4.
