# Supplementary material for: Quercetin 3,5,7,3′,4′-pentamethyl ether from Kaempferia parviflora directly and effectively activates human SIRT1
Source: Commun Biol. 2021 Feb 19;4:209. doi: 10.1038/s42003-021-01705-1 (PMC7896056; doi:10.1038/s42003-021-01705-1)
Supplement: Supplementary file 7 — Reporting Summary [file 42003_2021_1705_MOESM7_ESM.pdf]

nature research

Corresponding author(s): Koji Nagata

Last updated by author(s): Dec 30, 2020

# Reporting Summary

Nature Research wishes to improve the reproducibility of the work that we publish. This form provides structure for consistency and transparency in reporting. For further information on Nature Research policies, see Authors & Referees and the Editorial Policy Checklist.

## Statistics

For all statistical analyses, confirm that the following items are present in the figure legend, table legend, main text, or Methods section.

| n/a | Confirmed | |
|---|---|---|
| ☐ | ☒ | The exact sample size (*n*) for each experimental group/condition, given as a discrete number and unit of measurement |
| ☐ | ☒ | A statement on whether measurements were taken from distinct samples or whether the same sample was measured repeatedly |
| ☐ | ☒ | The statistical test(s) used AND whether they are one- or two-sided<br>*Only common tests should be described solely by name; describe more complex techniques in the Methods section.* |
| ☒ | ☐ | A description of all covariates tested |
| ☐ | ☒ | A description of any assumptions or corrections, such as tests of normality and adjustment for multiple comparisons |
| ☐ | ☒ | A full description of the statistical parameters including central tendency (e.g. means) or other basic estimates (e.g. regression coefficient) AND variation (e.g. standard deviation) or associated estimates of uncertainty (e.g. confidence intervals) |
| ☐ | ☒ | For null hypothesis testing, the test statistic (e.g. *F*, *t*, *r*) with confidence intervals, effect sizes, degrees of freedom and *P* value noted<br>*Give P values as exact values whenever suitable.* |
| ☒ | ☐ | For Bayesian analysis, information on the choice of priors and Markov chain Monte Carlo settings |
| ☒ | ☐ | For hierarchical and complex designs, identification of the appropriate level for tests and full reporting of outcomes |
| ☒ | ☐ | Estimates of effect sizes (e.g. Cohen's *d*, Pearson's *r*), indicating how they were calculated |

*Our web collection on statistics for biologists contains articles on many of the points above.*

## Software and code

Policy information about availability of computer code

| | |
|---|---|
| Data collection | ITC assay was performed using MicroCal iTC200 isothermal titration calorimeter (GE Healthcare) and the dissociation constant (KD) values were calculated using Origin 7.0 software provided by GE Healthcare. NMR data were collected using VNMRJ 4.2 NMR software. FRET data were collected using Spectra Manager Version 2.14.06 software. All MD simulations were performed with Gromacs 2019. |
| Data analysis | NMR spectra were processed with NMRPipe and analyzed using Sparky. |

For manuscripts utilizing custom algorithms or software that are central to the research but not yet described in published literature, software must be made available to editors/reviewers. We strongly encourage code deposition in a community repository (e.g. GitHub). See the Nature Research guidelines for submitting code & software for further information.

## Data

Policy information about availability of data

All manuscripts must include a data availability statement. This statement should provide the following information, where applicable:

- Accession codes, unique identifiers, or web links for publicly available datasets
- A list of figures that have associated raw data
- A description of any restrictions on data availability

There is no restriction on the data availability. All the data are available upon request to the corresponding author.

# Field-specific reporting

Please select the one below that is the best fit for your research. If you are not sure, read the appropriate sections before making your selection.

☒ Life sciences ☐ Behavioural & social sciences ☐ Ecological, evolutionary & environmental sciences

For a reference copy of the document with all sections, see nature.com/documents/nr-reporting-summary-flat.pdf

# Life sciences study design

All studies must disclose on these points even when the disclosure is negative.

| | |
|---|---|
| Sample size | Two SIRT1 constructs and two kinds of activators were used for the ITC assay. 15N-labeled SIRT1 NTD alone, supplemented with KPMF-8 or resveratrol were used for the NMR analysis. One SIRT1 construct was used for the FRET analysis. One kind of cell line and two kinds of activators were used for the HDAC assay. |
| Data exclusions | In ITC assay, the heat exchange in the first titration was excluded for calculating the KD value. There were no data exclusions in the NMR and FRET analyses and the HDAC assay. |
| Replication | All attempts for replication were successful. |
| Randomization | Allocations in this study are random. |
| Blinding | This study does not involve animals or human research, and blinding is not applicable to this type of research. |

# Reporting for specific materials, systems and methods

We require information from authors about some types of materials, experimental systems and methods used in many studies. Here, indicate whether each material, system or method listed is relevant to your study. If you are not sure if a list item applies to your research, read the appropriate section before selecting a response.

## Materials & experimental systems

| n/a | Involved in the study | |
|---|---|---|
| ☐ | ☒ | Antibodies |
| ☐ | ☒ | Eukaryotic cell lines |
| ☒ | ☐ | Palaeontology |
| ☒ | ☐ | Animals and other organisms |
| ☒ | ☐ | Human research participants |
| ☒ | ☐ | Clinical data |

## Methods

| n/a | Involved in the study | |
|---|---|---|
| ☒ | ☐ | ChIP-seq |
| ☒ | ☐ | Flow cytometry |
| ☒ | ☐ | MRI-based neuroimaging |

## Antibodies

| | |
|---|---|
| Antibodies used | antibodies against SIRT1 (#2310), β-actin (#4967) and anti-rabbit IgG, HRP-linked (Cell Signaling Technology) |
| Validation | Rabbit antibody against human SIRT1 (#2310): SirT1 antibody detects endogenous levels of total SirT1 protein. The antibody does not cross-react with other sirtuin proteins.<br>Rabbit antibody against β-actin (#4967) : β-Actin Antibody detects endogenous levels of β-actin. This antibody may cross-react with the γ-actin (cytoplasmic) isoform. It does not cross-react with α-skeletal, α-cardiac, α-vascular smooth, or γ-enteric smooth muscle isoforms. |

## Eukaryotic cell lines

Policy information about cell lines

| | |
|---|---|
| Cell line source(s) | MCF-7 human breast cancer cell line (ATCC No. CRL8305), a kind gift from Dr. Yoichi Hayakawa (Tokyo University of Science, Tokyo, Japan) |
| Authentication | Based on source authentication |
| Mycoplasma contamination | The cell line was tested negative for Mycoplasma contamination by PCR. |
| Commonly misidentified lines (See ICLAC register) | N/A |
